# Increased single-strand annealing rather than non-homologous end-joining predicts hereditary ovarian carcinoma

**DOI:** 10.18632/oncotarget.21720

**Published:** 2017-10-09

**Authors:** Miriam Deniz, Tatiana Romashova, Sarah Kostezka, Anke Faul, Theresa Gundelach, Maria Moreno-Villanueva, Wolfgang Janni, Thomas W.P. Friedl, Lisa Wiesmüller

**Affiliations:** ^1^ Department of Obstetrics and Gynecology, Ulm University, Ulm, Germany; ^2^ Department of Biology, University of Konstanz, Konstanz, Germany

**Keywords:** early-onset ovarian cancer, ovarian cancer risk, error-prone DNA repair, PARP activity, functional biomarker

## Abstract

Mutations in genes encoding DNA double-strand break (DSB) repair components, especially homologous recombination (HR) proteins, were found to predispose to breast and ovarian cancer. Beyond high penetrance risk gene mutations underlying monogenic defects, low risk gene mutations generate polygenic defects, enlarging the fraction of individuals with a predisposing phenotype. DSB repair dysfunction opens new options for targeted therapies; poly (ADP-ribose) polymerase (PARP) inhibitors have been approved for BRCA-mutated and platinum-responsive ovarian cancers. In this work, we performed functional analyses in peripheral blood lymphocytes (PBLs) using a case-control design. We examined 38 women with familial history of breast and/or ovarian cancer, 40 women with primary ovarian cancer and 34 healthy controls. Using a GFP-based test we analyzed error-prone DSB repair mechanisms which are known to compensate for HR defects and to generate chromosomal instabilities. While non-homologous end-joining (NHEJ) did not discriminate between cases and controls, we found increases of single-strand annealing (SSA) in women with familial risk vs. controls (P=0.016) and patients with ovarian cancer vs. controls (P=0.002). Consistent with compromised HR we also detected increased sensitivities to carboplatin in PBLs from high-risk individuals (P<0.0001) as well as patients (P=0.0011) compared to controls. Conversely, neither PARP inhibitor responses nor PARP activities were altered in PBLs from the case groups, but PARP activities increased with age in high-risk individuals, providing novel clues for differential drug mode-of-action. Our findings indicate the great potential of detecting SSA activities to deliver an estimate of ovarian cancer susceptibility and therapeutic responsiveness beyond the limitations of genotyping.

## INTRODUCTION

Ovarian cancer is the fifth most common female cancer in the western world, and the deadliest gynecological malignancy. The overall poor prognosis for ovarian cancer patients is a consequence of aggressive biological behavior and a lack of adequate diagnostic tools for early detection. In fact, approximately 70% of all patients with epithelial ovarian cancer are diagnosed at advanced tumor stages [1]. Beside reproductive, demographic, and lifestyle factors affecting the risk of ovarian cancer, the most important single risk factor is a familial history of hereditary breast and ovarian cancer cases [1–3]. *BRCA1* and *BRCA2* are the most frequently mutated genes in familial ovarian carcinoma [4, 5]. They play a key role in DSB repair by HR. Additionally, other genes involved in HR including *BRIP1*, *RAD51C* and *RAD51D* have been implicated in genetic susceptibility to ovarian carcinoma [6–9]. Beside germline also somatic gene mutations cause HR deficiency in ovarian cancer [5, 10]. HR is an important and the most accurate pathway for restoring DSBs. HR dysfunction leads to an increase in other less precise DSB repair mechanisms like SSA [11, 12]. This in turn can result in chromosomal instability with malignant transformation and is known to be crucial in the development of hereditary breast and ovarian cancer. Increases of canonical NHEJ activities, in particular, have been reported to correlate with and contribute to the phenotype of Fanconi Anemia (FA) caused by bi-allelic mutations in a set of DNA repair genes including genes like *BRCA2*, *PALB2* and *RAD51C* causing breast and/or ovarian cancer susceptibility in heterozygous mutation carriers [13, 14].

There are efforts made to further characterize breast and ovarian cancer beyond the limitations of genotyping by detecting and quantifying DNA repair activities [15–22]. Using a GFP-based test system for the analysis of distinct DSB repair pathways, we previously showed that de-repression of error-prone microhomology-mediated end joining (MMEJ) and SSA can be detected in lymphoblastoid cells (LCLs) from individuals with breast cancer predisposing *BRCA1*, *BRCA2* or *PALB2* mutations [23–25]. Furthermore, we found increases of MMEJ and SSA in primary peripheral blood lymphocytes (PBLs) from breast cancer patients and women with familial risk compared to healthy controls; in addition, elevated SSA was associated with young age (<50) at initial diagnosis of breast cancer, which could be indicative of genetic predisposition [19]. The presence of germline mutations and somatic changes causing HR dysfunction is called BRCAness and is predictive for primary sensitivity to platinum-based drugs and improved overall survival, similar to tumors with known *BRCA1/2* mutations [5, 26]. Currently *BRCA1/2* mutation carriers have access to targeted therapy with PARP inhibitors [22]. HR deficiency might predict therapy responsiveness beyond *BRCA1/2* mutations; in a recently published phase II trial patients with metastatic prostate cancer and aberrations in DNA repair genes including *BRCA1/2*, *ATM*, *FA* genes and *CHEK2* had a highly significantly increased therapeutic response to the PARP inhibitor olaparib compared to patients without such mutations [27]. These findings suggest that routinely performed tests for qualitative and quantitative assessment of DNA repair dysfunction might be used for risk assessment as well as for prediction of responsiveness to conventional therapy regimens involving platinum-based drugs and targeted therapies with PARP inhibitors.

In this work, we aimed to determine the status of selected DSB repair-related functions in PBLs from ovarian cancer patients and predisposed individuals using a case-control design. We analyzed distinct DSB repair pathways by the GFP-based test system established for pathogenic breast and ovarian risk gene mutations. In parallel, we examined sensitivities to platinum-based and PARP inhibitor therapy as well as PARP activities *ex vivo*. Our results suggest that *ex vivo* life cell functional analysis may close the gap between susceptibility gene sequencing and pedigree analysis on the way towards a comprehensive marker system for assessment of ovarian cancer risk.

## RESULTS

### Analysis of error-prone DSB repair pathways in PBLs from high risk family members, ovarian cancer patients and healthy women

**Figure 1:**
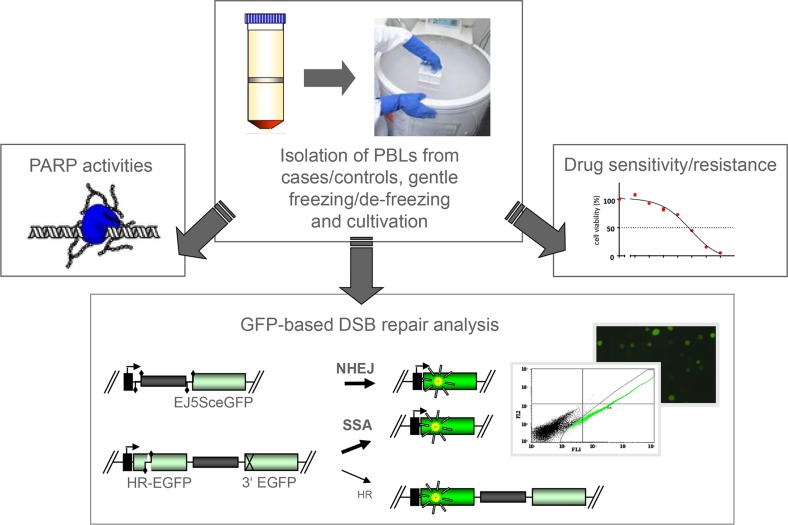
Outline of the study design After retrieval of heparinized blood samples from donors (38 high risk individuals, 40 breast cancer patients and 34 healthy controls) PBLs were isolated by Ficoll gradient centrifugation within 24h [19]. PBLs were transferred to liquid nitrogen and gently thawed according to the SOPs developed for the MARK-AGE EU project [28]. While PARP activities were determined in PBL aliquots immediately after de-freezing, the majority of cells were cultivated for 72h. For NHEJ and homologous repair (mostly SSA) measurements PBLs were nucleofected with the GFP-based DSB repair substrates EJ5SceGFP [29] and HR-EGFP/3´EGFP [69], respectively, and DSB repair was quantified by FACS analysis of the fraction of green fluorescent cells (corrected for individual transfection efficiencies) 24h later. I-*Sce*I-mediated cleavage at two recognition sites 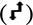 initiated NHEJ deleting the spacer sequence (grey bar) between the promoter (kinked arrow) and GFP coding region (green bar) in EJ5SceGFP. Cleavage within internally mutated HR-EGFP triggered homologous repair with N-terminally mutated 3´EGFP in substrate HR-EGFP/3´EGFP. Remaining PBLs were subjected to the analysis of drug sensitivities using MTT assay, whereby IC50-values were determined after carboplatin and PARP inhibitor (IQD) treatment.

To test whether error-prone and therefore detrimental DSB repair changes are associated with ovarian cancer, we performed functional analyses of blood-derived cells from 40 patients with primary diagnosis of ovarian cancer and 34 female, healthy, age-matched controls without previous cancer or familial history. To compare potential phenotypic changes of these sporadic ovarian cancer patients with those from members of families with increased breast and ovarian cancer risk, we further recruited 38 high-risk family members for functional blood sample analysis (Table 1, Supplementary Table 1). Blood samples were collected individually for each proband, PBLs isolated, gently frozen and thawed following the MARK-AGE SOP [28] before *ex vivo* culture and functional testing (Figure 1). Aliquots of a large batch of PBLs from a single healthy blood donor served as internal reference during all functional testings.

**Table 1:** Mean age of study participants tested for PARP activities, DSB repair activities and chemosensitivities: Cases and controls of the same age range

Age	High-risk individuals		Control cohort high risk individuals_*a*_			Patients		Control cohort patients_*b*_
	n	Mean age (SD)	n	Mean age (SD)	n	Mean age (SD)	n	Mean age (SD)
**NHEJ age distribution**	28	44.4 (8.2)	18	51.9 (7.5)	19	59.6 (12.6)	27	58.5 (11.5)
30-39	9 (32%)		1 (6%)		1 (5%)		1 (4%)	
40-49	11 (39%)		7 (39%)		4 (21%)		7 (26%)	
50-59	7 (25%)		6 (33%)		7 (37%)		6 (22%)	
60-69	1 (4%)		4 (22%)		2 (11%)		6 (22%)	
70-79	0 (0%)		0 (0%)		3 (16%)		6 (22%)	
>=80	0 (0%)		0 (0%)		2 (11%)		1 (4%)	
**SSA age distribution**	37	45.5 (7.9)	20	52.8 (7.7)	30	61.0 (12.1)	32	60.4 (12.0)
30-39	9 (24%)		1 (5%)		1 (3%)		1 (3%)	
40-49	17 (46%)		7 (35%)		5 (17%)		7 (22%)	
50-59	10 (27%)		6 (30%)		11 (37%)		6 (19%)	
60-69	1 (3%)		6 (30%)		4 (13%)		9 (28%)	
70-79	0 (0%)		0 (0%)		7 (23%)		7 (22%)	
>=80	0 (0%)		0 (0%)		2 (7%)		2 (6%)	
**PARP activity age distribution**	38	45.6 (7.7)	19	52.4 (7.6)	39	61.6 (13.3)	32	61.1 (12.2)
30-39	9 (24%)		1 (5%)		2 (5%)		1 (3%)	
40-49	16 (42%)		7 (37%)		7 (18%)		6 (19%)	
50-59	12 (32%)		6 (32%)		11 (28%)		6 (19%)	
60-69	1 (3%)		5 (26%)		5 (13%)		9 (28%)	
70-79	0 (0%)		0 (0%)		11 (28%)		8 (25%)	
>=80	0 (0%)		0 (0%)		3 (8%)		2 (6%)	
**IQD sensitivity age distribution**	25	45.6 (7.0)	15	53.9 (6.5)	23	61.8 (13.3)	25	61.4 (10.6)
30-39	6 (24%)		0 (0%)		1 (4%)		0 (0%)	
40-49	11 (44%)		5 (33%)		5 (22%)		5 (20%)	
50-59	8 (32%)		6 (40%)		5 (22%)		6 (24%)	
60-69	0 (0%)		4 (27%)		4 (17%)		7 (28%)	
70-79	0 (0%)		0 (0%)		6 (26%)		6 (24%)	
>=80	0 (0%)		0 (0%)		2 (9%)		1 (4%)	
**Carboplatin sensitivity age distribution**	29	45.2 (8.1)	16	53.0 (7.3)	26	61.3 (13.6)	27	61.0 (11.6)
30-39	8 (28%)		1 (6%)		2 (8%)		1 (4%)	
40-49	12 (41%)		5 (31%)		5 (19%)		5 (19%)	
50-59	8 (28%)		6 (38%)		5 (19%)		6 (22%)	
60-69	1 (3%)		4 (25%)		5 (19%)		7 (26%)	
70-79	0 (0%)		0 (0%)		7 (27%)		7 (26%)	
>=80	0 (0%)		0 (0%)		2 (8%)		1 (4%)	

_*a*_For the comparison between high-risk individuals and controls, a control subgroup was analyzed encompassing the corresponding age group of >=30 and <65 years.

_*b*_For the comparison between patients and controls, a control subgroup was analyzed encompassing the corresponding age group of >35 years.

**Figure 2:**
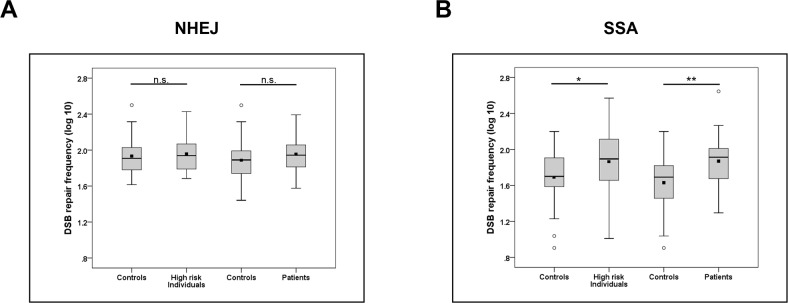
Association of elevated SSA frequencies with case status DSB repair, i.e. **(A)** NHEJ and **(B)** SSA, frequencies were determined in triplicates per individual in high-risk individuals (NHEJ: n=28, SSA: n=37) versus controls (NHEJ: n=18, SSA=20; corresponding age subgroup of >=30 and <65 years) and ovarian cancer patients (NHEJ: n=19, SSA: n=30) versus controls (NHEJ: n=27, SSA=32; corresponding age subgroup of >35 years). Mean values were individually corrected for nucleofection efficiencies (determined in triplicates), normalized to data from reference PBLs (internal standard for experimental day) and log_10_ transformed for normal distribution after transformation. For further details see Table 2. ^*^P<0.05; ^**^P<0.01; n.s., non-significant.

To analyze pathway-specific DSB repair we introduced the GFP-based reporter plasmids EJ5SceGFP and HR-EGFP/3´EGFP together with expression plasmid for I-*Sce*I meganuclease via nucleofection into PBLs (Figure 1). These extrachromosomal repair substrates were previously demonstrated to monitor NHEJ and homologous repair (mostly SSA) in PBLs following I-*Sce*I-mediated cleavage [19, 29, 30]. When comparing NHEJ activities in PBLs from high-risk individuals and controls of the corresponding age group (≥30 and <65 years) or from ovarian cancer patients and controls of the corresponding age group (>35 years) we did not find statistically significant differences. However, our analyses revealed significant differences of SSA frequencies in PBLs from both case groups as compared with the corresponding control groups with mean SSA values being 1.7-fold higher in high-risk individuals versus controls (*P*=0.016) and in ovarian cancer patients versus controls (*P*=0.002) (Table 2, Figure 2). These results from the univariate analysis of log_10_-transformed mean values per individual were confirmed by a general linear model analysis additionally correcting for the proband´s age.

**Table 2:** Differences between the mean pathway-specific DSB repair frequencies of high-risk individuals versus controls and ovarian cancer patients versus controls

	High-risk individuals		Controls _*a*_			
**DSB repair pathway**	**n**	**Mean (SD) log_10_**	**n**	**Mean (SD)log_10_**	**Difference of the means (95% CI)**_***b***_	***P* value**_***c***_
**NHEJ**	28	1.96 (0.21)	18	1.88 (0.19)	0.08 (-0.44-0.21)	0.198
**SSA**	37	1.86 (0.32)	20	1.64 (0.33)	0.22 (0.04-0.41)	0.016
	**Patients**		**Controls**			
**DSB repair pathway**	**n**	**Mean (SD) log_10_**	**n**	**Mean (SD)log_10_**	**Difference of the means (95% CI)**	***P* value**
**NHEJ**	19	1.95 (0.19)	27	1.89 (0.23)	0.07 (-0.06-0.19)	0.302
**SSA**	30	1.87 (0.27)	32	1.63 (0.32)	0.24 (0.09-0.39)	0.002

_*a*_ DSB repair frequencies were individually adjusted for the internal standard, i.e. the DSB repair frequency obtained with the reference blood sample analyzed on the same experimental day (set to 100%, which equals absolute mean DSB repair frequencies for NHEJ: 10.5×10^−2^ and for SSA: 1.4×10^−2^). Note that for the comparison between the case groups (high-risk individuals, patients) and controls, control subgroups were analyzed encompassing the corresponding age group each (controls compared with high-risk individuals: age >=30 and <65; controls compared with patients: age >35; for details of subgroups see Table 1).

_*b*_ CI = confidence interval.

_*c*_ *P* values for the t-test for equality of means were calculated for log_10_ transformed mean values per individual.

### Predictive power of SSA frequency rise

**Figure 3:**
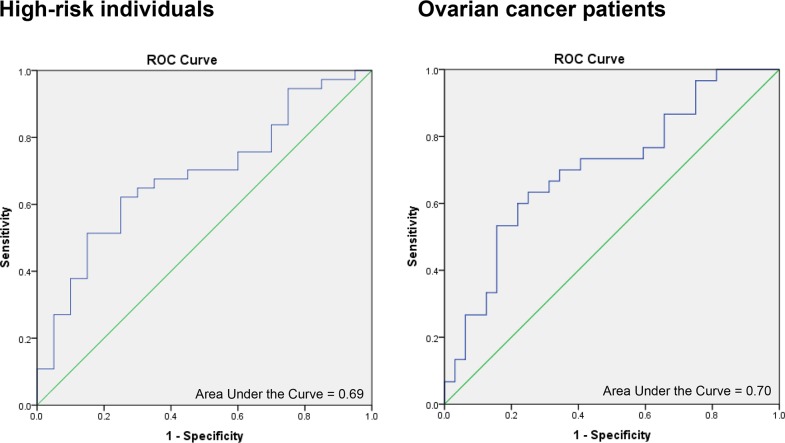
ROC curves for SSA values in cases versus controls ROC curves of SSA values for cases and controls, adjusted for internal standard and log_10_ transformed. **(A)** ROC curve for high-risk individuals (n=37) and controls (n=20). Area under the curve: 0.69, *P*=0.022. **(B)** ROC curve for ovarian cancer patients (n=30) and controls (n=32). Area under the curve: 0.70, *P*=0.006.

Having observed elevated SSA in PBLs from the case groups, we next estimated the power of this DSB repair phenotype to predict allocation of women to the high-risk or ovarian cancer group. The results from calculating ORs using binary logistic regression analysis adjusted for age and the areas under the ROC curves (AUC values) for univariate discrimination between cases and controls are presented in Table 3 and Figure 3. ORs refer to a unit change for DSB repair frequencies, with units being log_10_ transformed DSB repair frequencies. As was expected from the lack of an association between NHEJ frequencies and case status, ORs or AUC values did not reach statistical significance for this DSB repair pathway. Conversely, the predictive power was highly significant for SSA frequencies scored in ovarian cancer patients versus controls with an OR of 19.16 (*P*=0.006) and an AUC value of 0.70 (*P*=0.006). For the comparison of high-risk group members and controls, we calculated an OR of 7.03, which was close to statistical significance (*P*=0.059), and an AUC value of 0.69 (*P*=0.022). Restricting the results from the group of high-risk individuals to the frequencies referring to family members with a known probability ≥20% for being a heterozygous carrier of a breast and ovarian cancer susceptibility allele (calculated via pedigree analysis using software Cyrillic 2.1.3), did not increase the predictive power. To more stringently evaluate the influence of genetic predisposition, we restricted the high-risk individual data set to the ten high-risk individuals with pathogenic or likely pathogenic *BRCA1* or *BRCA2* mutations (i.e. class 5 or 4 according to the guidelines of the German multicenter consortium for Hereditary Mammary and Ovarian Carcinoma and in analogy to the International Agency for Research on Cancer, IARC). A ROC curve analysis with this restricted high-risk individual data set versus controls showed an increased AUC value of 0.74 (*P*=0.035) (Supplementary Figure 1A). Thus, the predictive power of elevated SSA was higher than for the original high-risk individual versus control comparison, when limiting to mutation carriers with pathogenic BRCA mutations. Our results therefore suggest an association of elevated SSA repair with ovarian cancer and hereditary breast and ovarian cancer risk linked to BRCAness.

**Table 3:** Predictive power of pathway-specific DSB repair activities for discrimination between high-risk individuals versus controls and ovarian cancer patients versus controls_*a*_

DSB repair pathway	High-riskindividualsn	Controls_*b*_n	OR_*c*_ (95% CI)	*P* value	Area under the ROC curve	*P* value
**NHEJ**	28	18	16.01 (0.46-563.17)	0.127	0.62	0.192
**SSA**	37	20	7.03 (0.93-53.13)	0.059	0.69	0.022
**DSB repair pathway**	**Patientsn**	**Controlsn**	**OR (95% CI)**	***P* value**	**Area under the ROC curve**	***P* value**
**NHEJ**	19	27	4.46 (0.25-79.77)	0.309	0.61	0.208
**SSA**	30	32	19.16 (2.33-157.28)	0.006	0.70	0.006

_*a*_ DSB repair frequencies were individually adjusted for the internal standard (100%), i.e. the DSB repair frequency obtained with the reference blood sample analyzed on the same experimental day.

_*b*_ As for the comparison between the case groups (high-risk individuals, patients) and controls, control subgroups were analyzed encompassing the corresponding age group each (controls compared with high-risk individuals: age >=30 and <65; controls compared with patients: age >35; for details of subgroups see Table 1).

_*c*_ The odds ratios (OR) per unit change for each pathway-specific DSB repair frequency given are based on age-adjusted binary logistic regression analysis. Units are log_10_-transformed DSB repair frequencies.

### Analysis of PARP activities in PBLs from cases and controls

Elevated PARP1 expression has been detected in several tumor types including breast and ovarian cancer with a further rise in triple-negative breast cancer and *BRCA1*- or *BRCA2*-mutated cancers [31, 32]. To determine the individual PARP status we applied an established protocol for measurements of cellular poly(ADP-ribosyl)ation (PARylation) capacities in PBLs using flow cytometry [33]. For assessment of activated (versus basal) PARP activities, which are known to unfold in response to DNA damage, we quantified PARylation under maximal stimulation by addition of excess NAD+ substrate and oligonucleotides mimicking single-stranded DNA breaks. Different from our findings for SSA, both basal and activated PARP activities in PBLs neither correlated with high-risk nor ovarian cancer case status as compared to control status (Table 4). Age-adjusted general linear model also did not carve out statistically significant differences (all *P*>0.05), but a trend for increased basal and activated PARP activities in high-risk individuals compared to controls (both *P*<0.10). Correspondingly, predictive power calculations demonstrated absence of statistical significances for ORs and AUC values indicating that neither basal nor activated PARP activities discriminate between case and control groups (Table 5).

**Table 4:** Differences between the mean PARP activities of high-risk individuals versus controls and ovarian cancer patients versus controls

	High-risk individuals		Controls _*a*_			
PARP activity	n	Mean (SD) log_10_	n	Mean (SD)log_10_	Difference of the means (95% CI) _*b*_	*P* value _*c*_
**Basal**	38	2.34 (0.25)	19	2.27 (0.24)	0.06 (-0.08-0.20)	0.364
**activated**	38	2.28 (0.28)	19	2.19 (0.30)	0.09 (-0.07-0.25)	0.281
	**Patients**		**Controls**			
**PARP activity**	**n**	**Mean (SD)log_10_**	**n**	**Mean (SD)log_10_**	**Difference of the means (95% CI)**	***P* value**
**Basal**	39	2.30 (0.30)	32	2.29 (0.23)	0.02 (-0.11-0.15)	0.798
**activated**	39	2.21 (0.30)	32	2.26 (0.28)	-0.05 (-0.19-0.09)	0.465

_*a*_ PARP activities were individually adjusted for the internal standard, i.e. the PARP activity obtained from the reference blood sample analyzed on the same experimental day (set to 100%, which equals absolute MFI mean values for basal PARP activity: 29.5×10^−2^ and for activated PARP activity: 78.4×10^−2^). Note that for the comparison between the case groups (high-risk individuals, patients) and controls, control subgroups were analyzed encompassing the corresponding age group each (controls compared with high-risk individuals: age >=30 and <65; controls compared with patients: age >35).

_*b*_ CI = confidence interval.

_*c*_ *P* values for the t-test for equality of means were calculated for log_10_ transformed mean values per individual.

**Table 5:** Predictive power of PARP activities for discrimination between high-risk individuals versus controls and ovarian cancer patients versus controls_*a*_

PARP activity	High-risk individualsn	Controls_*b*_n	OR_*c*_ (95% CI)	*P* value	Area under the ROC curve	*P* value
**Basal**	38	19	8.34 (0.61-114.47)	0.112	0.56	0.446
**activated**	38	19	5.57 (0.62-50.04)	0.125	0.59	0.257
**PARP activity**	**Patientsn**	**Controlsn**	**OR (95% CI)**	***P* value**	**Area under the ROC curve**	***P* value**
**Basal**	39	32	1.23 (0.21-7.06)	0.817	0.51	0.890
**activated**	39	32	0.48 (0.09-2.61)	0.391	0.48	0.720

_*a*_ PARP activities were individually adjusted for the internal standard (100%), i.e. the PARP activity obtained with the reference blood sample analyzed on the same experimental day.

_*b*_ As for the comparison between the case groups (high-risk individuals, patients) and controls, control subgroups were analyzed encompassing the corresponding age group each (controls compared with high-risk individuals: age >=30 and <65; controls compared with patients: age >35).

_*c*_ The odds ratios (OR) per unit change for each pathway-specific DSB repair frequency given are based on age-adjusted binary logistic regression analysis. Units are log_10_–transformed DSB repair frequencies.

**Figure 4:**
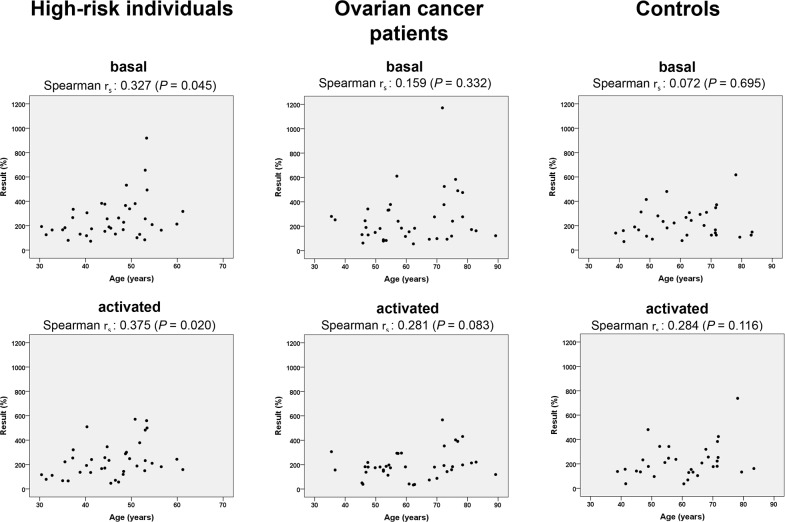
PARP activities as a function of age PARP activities (basal and following oligonucleotide plus NAD+ activation) were determined in thawed PBL samples, normalized to reference PBL values and graphically presented as a function of each proband´s age (high-risk individuals: n=38; ovarian cancer patients: n=39; controls: n=32). Spearman´s rho (r_s_) correlation coefficient and two-tailed significance were calculated as indicated.

Activation of PARP1 represents a crucial component of the DNA damage response, with involvement in various mechanisms of DNA repair, and has been linked with lifespan determination [34, 35]. When we analyzed PARP activities in PBLs as a function of age via Spearman correlation test, we observed significant increases of both basal (*P*=0.045) and activated (*P*=0.020) activities with increasing age of women from the high-risk group (Figure 4). No age-dependency of PARP activities was seen in PBLs from control individuals. In ovarian cancer patients, a trend of increasing activated PARP activities (*P*=0.083) was detectable, which was not noticeable for basal PARP activities. In conclusion, PARP activities were not associated with ovarian cancer or hereditary breast and ovarian cancer risk but were correlated with age in high-risk individuals.

### *Ex vivo* determination of PARP inhibitor and carboplatin treatment responses

**Figure 5:**
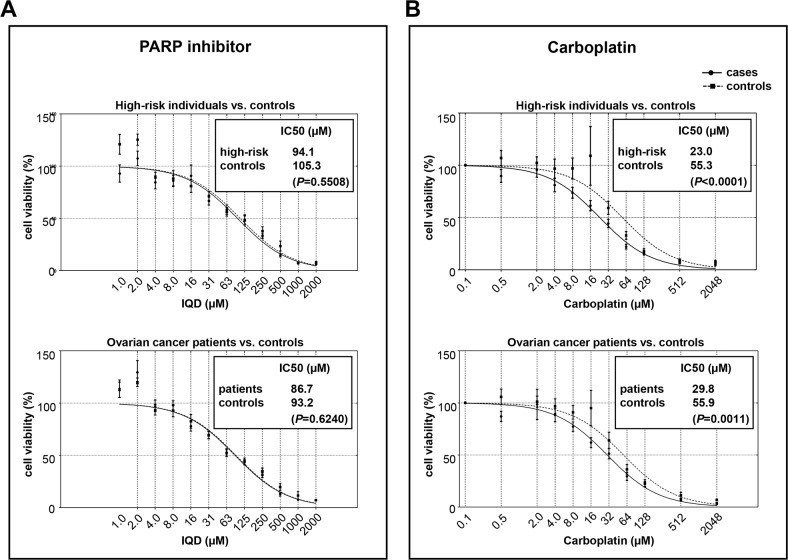
Drug sensitivities in cases versus controls Cell viabilities were assessed by use of MTT assay after 7d of PARP inhibitor (IQD) treatment **(A)** and 48h of carboplatin treatment **(B)**, respectively. Mean survival curves of PBLs derived from high-risk individuals (IQD: n=25; carboplatin: n=29) versus controls from the corresponding age subgroup of >=30 and <65 years (IQD: n=15; carboplatin: n=16) and ovarian cancer patients (IQD: n=23; carboplatin: n=26) versus controls from the corresponding age subgroup of >35 years (IQD: n=25; carboplatin: n=27) are graphically presented. Calculation of IC50 values and statistical tests for differences between survival curves were performed using GraphPad Prism software.

Recently, various PARP inhibitors have entered the clinic for treatment of BRCA-mutated and platinum-responsive ovarian cancers [36]. Changes in various DNA repair genes in tumor biopsies were further demonstrated to correlate with clinical PARP inhibitor responses in metastatic prostate cancer patients [27]. In our previous studies, we demonstrated increased sensitivity to PARP inhibitor 1,5-isoquinolinediol (IQD) in immortalized lymphocytes (LCLs) with homozygously mutated *BRCA2* and in mammary epithelial cells with homozygously mutated *BRCA1* [21, 25]. Therefore, we asked whether DSB repair defects in our case groups as indicated by SSA increases also translate into altered responses to the PARP inhibitor IQD in PBLs (Figure 5). Assessment of cell viabilities using MTT assay and graphic presentation of the mean values obtained for each drug concentration from the different individuals per group revealed overlapping survival curves for the results from high-risk individuals and corresponding controls as well as from patients and controls. Consistently, IC50 values calculated from these curves were not significantly different (Figure 5A), which was also true when comparing the means of individually determined IC50 values (Supplementary Table 2).

Clinical trials have provided evidence for a high level of sensitivity to platinum derivatives in tumors with hereditary breast and ovarian cancer risk gene mutations [37]. Determination of cell viabilities in response to increasing concentrations of carboplatin and comparison of the resulting mean survival curves revealed increased sensitivities of PBLs from high-risk individuals (IC50=23.0μM) versus corresponding controls (IC50=55.3 μM) and ovarian cancer patients (IC50=29.8 μM) versus controls (IC50=55.9 μM) with high statistical significance (*P*≤0.0011) (Figure 5B). Cohort comparison of the mean IC50 values calculated for each individual supported the finding of elevated carboplatin sensitivity in high-risk individuals versus controls (P=0.035). Predictive power calculations further demonstrated statistical significances for ORs and AUC values, indicating that changes in the IC50 values discriminate between the high-risk and control groups (Supplementary Table 2 and 3). Separate analysis of the mean survival curves for PBLs from high-risk individuals with defined *BRCA1* and/or *BRCA2* status (n=6) revealed the lowest IC50 value for *BRCA1* and *BRCA2* mutation (class4-5) carriers (IC50=16.8 μM) versus controls (n=16) with high statistical significance (P=0.0021) and a trend towards higher sensitivity versus *BRCA1* and *BRCA2* variant (class1-3) carriers (n=15, P=0.0842) (Supplementary Figure 1B).

However, corresponding cohort comparison failed to confirm sensitization in ovarian cancer patients versus controls (P=0.126) (Supplementary Table 2). Since our prior case-control study revealed increased SSA frequencies in PBLs from young versus middle-aged breast cancer patients [19], we stratified the groups of ovarian cancer patients and controls accordingly. Notably, when focusing on the group of young individuals (<50 years) we observed a significantly reduced mean IC50 value in patients compared with controls (*P*=0.046) (Supplementary Figure 2). Statistically significant differences between patients and controls from the middle-aged (50-69 years) and aged (>70) patient versus control groups were not detectable. Of note, SSA frequencies and carboplatin sensitivities (IC50 values) did not reveal a statistically significant correlation when evaluating doubly tested PBLs from high-risk individuals (n=29) and ovarian carcinoma patients (n=17) using the non-parametric Spearman rank correlation coefficient r_s_ (data not shown), suggesting that carboplatin sensitivity is not tightly connected with SSA. Based on these results, we conclude that sensitivity to PARP inhibitory drug treatment did not discriminate between case and control groups, whereas increased carboplatin sensitivity was associated with hereditary breast and ovarian cancer risk as well as early-onset ovarian cancer.

## DISCUSSION

In this case control study associations between different DSB repair-related cellular functions and ovarian cancer (risk) were evaluated using PBLs. Our results showed increased SSA in ovarian cancer patients and predisposed women compared to healthy controls, i.e. an error-prone homologous DSB repair pathway which can compensate for inefficient HR. Consistent with a BRCAness-like, hereditary DSB repair defect, PBLs from high-risk individuals and young ovarian cancer patients (<50) were more sensitive to carboplatin treatment compared to controls. In contrast, neither NHEJ, PARP activities nor PARP inhibitor sensitivities were associated with case status. Our data suggest that the detection of elevated SSA in PBLs (and possibly *ex vivo* determination of carboplatin sensitivity) may serve as a biomarker contributing to the assessment of ovarian cancer risk. New predisposing genetic alterations are constantly emerging, increasingly more often without sufficient information on the pathogenic effect from pedigree analysis. Moreover, these genetic changes include risk gene variants of intermediate or low penetrance, which however can modify cancer risk in combination with other variants [38–42]. Known breast and ovarian cancer risk and modifier genes cluster in DSB repair pathways [43], but indirect effects such as on DSB repair gene expression, post-transcriptional mRNA processing and editing have to be considered as well [44–46]. Phenotypic marker systems based on functionally testing blood-derived cells may address these unmet challenges, i.e. may capture the multifaceted sources of ovarian cancer risk.

Analysis of the predictive power of SSA frequencies revealed a similar AUC value and an even higher OR to predict allocation into the group of ovarian cancer patients as compared with allocation into the group of high-risk individuals. In our previous work, we determined SSA activities in PBLs from sporadic breast cancer patients and high-risk individuals versus controls using the same SSA substrate [19]. From these earlier data we calculated both a higher AUC value and OR for the discrimination of high-risk individuals versus controls as compared to breast cancer patients versus controls. This was true even though the mean ages of breast and ovarian cancer patients were the same in the two case-control studies (61 years). Moreover, the calculated risk for being a heterozygous carrier of a susceptibility allele in the previous and newly analyzed group of high-risk individuals were very similar, as the probability was ≥20% in 86% and 83% of the cases, respectively. At first sight, this observation might indicate that ovarian versus breast cancer patients are more frequently affected by hereditary DSB repair defects. Another explanation could be that ovarian and breast cancer risk gene phenotypes may slightly differ with regard to de-repression of SSA versus other error-prone DSB repair processes. In support of this idea, we previously noticed that cells with defined predisposing mutations differ regarding the ratio of SSA versus MMEJ changes, even for different mutations within the same gene [19, 25]. Mechanistically these differences could be due to the stage within the HR pathway at which the particular gene product acts and at which distinct mechanisms rescue DSB repair [47, 48].

Our DSB repair analyses further suggested that quantification of NHEJ neither discriminated between predisposed individuals and controls nor ovarian cancer patients and healthy women. Here, we used reporter plasmid EJ5SceGFP, which was designed to measure both canonical and non-canonical NHEJ [29]. Use of the same reporter previously demonstrated elevated NHEJ in patient cells with homozygous FA gene mutation [49]. In LCLs from heterozygous carriers of a FA subtype N (*FancN*/*PALB2*)-mutation, which increases breast cancer risk six-fold [50], we previously found a relative increase of total NHEJ, but to a significantly lesser extent than SSA [25]. Our earlier studies rather demonstrated up-regulation of MMEJ, i.e. a NHEJ sub-pathway, in cells carrying various defined mutations in breast cancer susceptibility genes and in high-risk family members independently of the genotype [19, 24, 25]. However, compared with SSA, MMEJ increases were found to be less robust resulting in a lower predictive value [19]. Canonical NHEJ is partially error-free and error-prone [51]. MMEJ and SSA are always error-prone, as they rejoin DNA ends via short and long internal homologies, respectively, resulting in the deletion of terminal sequences [47]. Nucleolytic processing of the DNA ends after formation of a DSB is the initial step of MMEJ, SSA and HR. Following end processing, HR engages the RAD51-dependent machinery for strand invasion, whereas the alternative pathway SSA relies on RAD52-mediated single-strand annealing. Intriguingly, we previously observed that in cells with a pathogenic *PALB2*-mutation RAD52 not only promotes SSA but also MMEJ [25]. Therefore, MMEJ and SSA are mechanistically related, whereas canonical NHEJ repairs DSBs before end processing and engages a different set of DNA repair proteins [47]. Strikingly, in none of our phenotypic characterization studies using cells from healthy carriers of heterozygous mutations in *BRCA1*, *BRCA2* or *PALB2*, we observed a significant decline of HR, which however, was detectable in FancD1 cells with bi-allelic *BRCA2* mutations. This was true for both, the use of GFP-based reporter assays as well as for the analysis of RAD51 filament assembly visualized by immunofluorescence microscopy of nuclear RAD51 foci [24, 25]. Therefore, we propose that cells from FA patients with bi-allelic mutations suffer from a severe failure to execute HR unleashing alternative repair pathways on persisting DSBs like SSA and MMEJ and even canonical NHEJ, which emanates further upstream. Cells from individuals with increased breast and ovarian cancer risk carrying heterozygous mutations in susceptibility genes including certain FA genes are still able to execute HR and the homologous repair machinery still competes with canonical NHEJ proteins [47]. Further downstream, once processing of the DNA ends takes place, cells from high-risk individuals display haplo-insufficiency regarding suppression of error-prone MMEJ and SSA, the two pathways which like HR use single-stranded DNA ends. In conclusion, de-regulation of SSA but not NHEJ reliably indicates subtle functional changes associated with ovarian cancer (risk).

Today, PARP inhibitors are well-established drugs targeting HR-defective tumor cells and were clinically approved for treatment of platinum-sensitive and BRCA-mutated ovarian cancer patients [36, 52]. In our study presented here, we did not obtain evidence for altered PARP inhibitor responsiveness in PBLs from high-risk individuals or ovarian cancer patients as compared to healthy controls. Previously, we observed a highly significant response in homozygously *BRCA1*- or *BRCA2*-mutated cells, but either failed to detect a significant change or determined marginally significantly reduced IC50 values in heterozygously *BRCA1*-, *BRCA2*- and *PALB2*- mutated cells [21, 25]. In agreement with our findings, clinical PARP inhibitor use was originally based on the concept that loss of the wild-type BRCA allele represents an obligatory step for specific tumor cell killing [53]. More recently, Fleury and colleagues [54] demonstrated that PARP inhibitor response extends beyond the BRCA status, as combined deficiencies in HR plus another DNA repair pathway culminate in full responsiveness of high-grade serous epithelial ovarian cancer cell lines. Our results showing lack of responsiveness in non-tumor cells are therefore in line with the acceptable side-effect profile of these drugs in the heterozygous BRCA-mutation carriers.

PARP1 is a key sensor of endogenously and exogenously induced DNA lesions, responds immediately with PARylation of various proteins and thereby regulates the DNA damage response and DNA repair at multiple levels [35]. Several reports proposed that PARP1 expression and/or PARylation activity might represent surrogate markers for compromised DNA repair such as BRCAness [31–33, 55–57]. Here, we investigated basal and maximal PARP activities rather than PARP1 protein to also capture PARylation by other PARP family members and activation by post-translational modifications. We did not detect any correlation between PARP activities and case status, which is compatible with lack of an association between PARP activity and BRCA1 promoter hypermethylation [55]. Elevated PARP1 protein expression in breast cancer specimens with BRCAness was reported, but conflicting results have been obtained regarding a potential correlation between PARP1 protein levels and PARP activities [31, 32, 57, 58]. In analogy to PARP inhibitor sensitivities it is conceivable that PARP activities may have to be tested in the severely HR dysfunctional tumor tissue rather than blood-derived cells with subtle HR changes. Alternatively, PARP activities may not be a sufficiently sensitive marker for this DNA repair defect as compared with expression analysis. Interestingly however, we did notice an increase particularly of activated PARP activities in high-risk individuals with age, with the same trend in ovarian cancer patients but not in the controls. Ageing is accompanied by a decline of DSB repair [59], which is predicted to be aggravated in high-risk individuals. The combined deficiencies from hereditary predisposition and ageing may thus generate a DNA damage response reaching the sensitivity level of PARP activity measurements in human PBLs. Notably, we previously observed increasing PARP inhibitor sensitivities and a deregulation of DSB repair in epithelial cells from breast cancer specimens with age [21]. In our study presented here, we did not find differences in PARP inhibitor sensitivities or DSB repair activities in high-risk individuals or ovarian cancer patients with age. Future studies are warranted to clarify whether cells from the mammary gland and the ovaries show a greater dependency on HR as compared to cells from the hematopoietic system and therefore are more susceptible to DNA damage accumulation during aging in human beings.

In contrast to our findings with PARP inhibitor, we monitored significantly elevated carboplatin sensitivities in high-risk individuals and ovarian cancer patients. Among the patients pronounced responsiveness was attributable to the group of young patients at age <50 years, which is in agreement with a higher number of DSB repair gene mutations in early onset ovarian cancer [60]. Therefore, *ex vivo* responsiveness to treatment with carboplatin seems to detect pathogenic defects in ovarian cancer susceptibility genes with a higher sensitivity than with PARP inhibitory drugs. Consistently and again in contrast to PARP inhibitor treatment, we also discriminated LCLs with heterozygous BRCA-mutation from wild-type controls when determining IC50 values after carboplatin treatment (data not shown). Platinum derivatives induce DNA cross-links, which block DNA replication and have to be removed in a complex series of DNA repair processes [61]. Platinum drug-induced cross-link repair generates DSB intermediates, which are known to be particularly toxic and thus to induce side-effects. These one-sided DSBs have to be removed by the HR pathway in specific, i.e. mechanisms like NHEJ cannot compensate for HR defects [49]. Carboplatin treatment therefore exerts a strong effect on HR-deficient cells. Of interest regarding the observed differences in carboplatin and PARP inhibitor responses, PARP1 not only modulates DNA repair processes but also plays a role in the protection of stalled DNA replication forks by promoting fork reversal [62]. Restart of stalled forks can be mediated by different mechanisms including fork reversal or HR [63]. In this context it is of interest that Schlacher and colleagues [64] showed that functions of BRCA2 in fork protection and HR can be separated, whereby PARP inhibitor sensitivity increases with loss of fork protection in particular. It is therefore conceivable that subtle HR defects in heterozygously BRCA-mutated cells can be tolerated in PARP inhibitor- but not carboplatin-treated cells.

Taken together, our findings demonstrate that increased SSA is detectable in blood-derived cells from women with ovarian cancer and familial risk. Our establishment of *ex vivo* SSA measurements using freshly isolated and stored frozen PBLs paves the way for multicenter validation and thus offers new strategies for individual cancer risk prediction. Notably, most other functional approaches including use of HR or NHEJ reporter, quantification of DNA breaks, damage marker or RAD51 foci, PARP activities as well as assessment of PARP inhibitor responses failed to carve out a phenotypic marker system for direct use in patient cells to capture polygenic mechanisms (1, 25, this work). SSA represents an error-prone DNA repair mechanism, which is predicted to generate genomic instabilities associated with BRCA-mutation status. Therefore SSA analysis monitors a central process underlying carcinogenesis in these high-risk individuals. Strikingly, we also found an increase of carboplatin sensitivity in PBLs from predisposed individuals. Whether increased SSA activities – like BRCA-mutation status - also predict responsiveness of cancer patients to carboplatin and PARP inhibitors remains to be determined in prospective clinical trials. Recent efforts to develop novel treatment strategies uncovered synthetic lethality of BRCA1- and BRCA2-dependent HR dysfunction with inactivation of the key SSA component RAD52 [65, 66]. This finding indicated that SSA represents an essential pathway upon HR dysfunction, which can be directly measured in blood-derived cells as shown in this work, thus opening new options for targeted treatment and predictive testing.

## MATERIALS AND METHODS

### Study population

The study population comprised 38 female members of families with defined history of familial breast and/or ovarian cancer (high-risk individuals), 40 primary ovarian cancer patients (patients), and 34 healthy women without any previous cancer and without any family history of breast and/or ovarian cancer (controls) (see Table 1 for further details). The recruitment took place at the Department of Obstetrics and Gynecology at Ulm University, Germany, from June 2012 to September 2014. Patients were recruited after primary diagnosis and before their surgical therapy. From March 2013 to April 2015 high-risk individuals included in this study were interviewed and counseled according to the criteria of the German multicenter consortium for Hereditary Mammary and Ovarian Carcinoma [67]. Hereditary breast and ovarian cancer risk was assessed via pedigree analysis using software Cyrillic 2.1.3 [68]. Genetic testing for *BRCA1* and *BRCA2* mutations was offered to women with defined family history of cancer and performed for 29 out of the 38 family members, indicating *BRCA1* or *BRCA2* mutations in 8 and 5 cases, respectively. Among the 35 individuals with known lifetime and heterozygote risk, 23 women had a lifetime risk with 30% or more (65.7%) and 29 women a heterozygote risk for pathogenic mutations of 20% or more (82.9%). From the remaining individuals with a heterozygote risk below 20%, one of them had a mutagenic *BRCA2*-mutation. Controls were healthy female citizens from southern Germany recruited in parallel. The study was approved by the ethics committee of Ulm University (approval #157/2010) and informed consent has been obtained from all study participants. High-risk individuals were all below 62 years and patients at least 38 years of age (Table 1). For comparison between high-risk individuals and controls, the control group was restricted to age >= 30 and <65, for comparison with patients it was restricted to age >35. Details of subgroups are shown in Supplementary Table 1. This procedure resulted in a similar mean age of particular sub-groups tested for DSB repair activities and chemosensitivities (high-risk individuals, controls) (Supplementary Table 1).

### Blood samples and cell cultures

Here, we newly addressed the challenge of standardized sample qualities for life cell analysis by use of a standard operating procedure (SOP) developed for the population-based, multicentric MARK-AGE EU project [28]. Preparatory side-by-side analysis assured comparable viabilities, nucleofection efficiencies, DSB repair capacities and qualities in de-frozen versus freshly isolated PBLs (data not shown). In specific, heparinized blood samples (20ml) were obtained by venepuncture. PBLs were isolated as described by Keimling et al. [19] by density gradient centrifugation using Ficoll-Paque-PLUS, (LSM 1077 Lymphocyte, GE Healthcare, Germany) followed by several washing steps in PBS to remove thrombocytes and stored overnight at −80°C in Mr. Frosty Nalgene freezing containers (Sigma) with freezing medium containing 20% RPMI 1640 (Gibco BRL Life Technologies, Eggenstein, Germany), 70% FBS (Biochrom GmbH, Berlin, Germany) plus 10% DMSO (Calbiochem-Merck) [28]. For de-freezing, cryovials were transferred to a 37°C water bath. A volume of 0.5ml 37°C warm thawing medium (90% RPMI plus 10% FBS) per 2ml frozen cell suspension was added dropwise. After one minute the cell suspension was transferred into a 15ml polypropylene tube and thawing medium added stepwise. After centrifugation cells were resuspended in PB-Max medium including 2% phytohemagglutinin (Gibco, Germany) and cultivated for 72h at 37°C [19]. On each experimental day PBLs from aliquots originating from one and the same healthy donor and blood draw were cultivated as internal standard in parallel.

### DSB repair analyses

DSB repair was analyzed by use of an established enhanced GFP (EGFP)-based test system as described earlier [19, 69]. In brief, PBLs were harvested by centrifugation. Then, different DNA mixtures, containing the expression plasmid for the endonuclease I-*Sce*I (pCMV-I-SceI) together with one of the DSB repair substrates (see Figure 1) and pBS filler plasmid (pBlueScriptII KS, Stratagene, Heidelberg, Germany) or wild-type EGFP expression plasmid (for determination of transfection efficiencies) were introduced by nucleofection according to the amaxa protocol (Lonza, Cologne, Germany). Depending on the yield of PBLs from the blood sample, we tested DSB repair using substrate HR-EGFP/3´EGFP only or both HR-EGFP/3´EGFP and EJ5SceGFP [29]. Subsequently, the cells were re-cultivated for 24h and harvested for FACS analysis. Reconstitution of wild-type EGFP served as a measure of successful repair and was monitored via FACS analysis-based quantification of the fraction of green fluorescent cells. Thus, 50 000-100 000 living cells (according to life gate in FSC/SSC dot plot) were examined per sample to distinguish between GFP-positive and GFP-negative cells by the diagonal gating method in the Fl1/Fl2 dot plot (FACS Calibur® FACScan, Becton Dickinson). Each quantification of green fluorescent cells in repair assays was normalized by use of the individually determined transfection efficiency (20-80%) to calculate the DSB repair frequency. Mean DSB repair frequencies per blood sample and DNA substrate were based on duplicate or triplicate measurements each. Reference PBLs from the same healthy donor and blood draw showed the following mean DSB repair frequencies +/-SD during 13 batch analyses (triplicates each): NHEJ=10.54×10^−2^+/-7.06, SSA=1.40×10^−2^+/-1.27, and transfection efficiencies: 40.37+/-8.66 x10^−2^.

### PARP activities

PARP activities were determined in PBL aliquots immediately after de-freezing, i.e. without cultivation of PBLs. We used the flow cytometry-based technique exactly following protocol B described by Kunzmann et al. [33] to assess basal levels of cellular PARP activity as well as the stimulation of PARP activity induced by addition of NAD+ and activator oligonucleotide. This methodology is useful for the determination of cellular PARylation capacity and allows the selective analysis of mononuclear cells by gating and detection of a possible heterogeneity in PARylation capacity between cells of the same type.

### Drug sensitivity and resistance

PARP inhibitor and carboplatin sensitivity were assessed by the colorimetric MTT assay as described [70]. PBLs were treated with PARP inhibitor 1,5-isoquinolinediol (IQD) (ENZO, New York, NY, USA) in concentrations from 1 μM to 2 mM for 7d replacing the medium by fresh IQD-containing medium twice. carboplatin was used in concentrations ranging from 0.1 μM to 2.048 μM replacing the medium after 24h with fresh carboplatin-free medium. Data sets were corrected for mock-treatment for each drug concentration. Cell viability curves were generated and IC50-values calculated using GraphpadPrism version 5.04 (La Jolla, USA).

### Statistical analyses

To improve homogeneity of variances and approach normal distributions, all results on DSB repair frequencies and PARP activities were log10 transformed. For all analyses, mean log10 transformed DSB repair frequencies and PARP activities per individual and substrate - based on duplicate or triplicate measurements each - were used.

Mean log10 transformed DSB repair frequencies and PARP activities were compared between high-risk individuals and controls, as well as between patients and controls by unpaired t tests. Confirmatory analyses were performed using General Linear Models adjusted for age. Comparisons among groups are illustrated using Box-and-Whisker plots, where the horizontal line inside the box represents the median, a black square denotes the mean, and the box indicates the interquartile range (IQR; the middle 50% of scores). The ends of the whiskers denote the lowest and highest values still within 1.5 IQR of the lower and upper quartile (i.e. the lower and upper end of the box), respectively. If there are no values more than 1.5 IQR below the lower or above the upper quartile (i.e. outliers), the ends of the whiskers denote minimum and maximum of the data. Outliers that are more than 1.5 IQR but less than 3 IQR below the lower or above the upper quartile are indicated by open circles, and extreme outliers more than 3 IQR below the lower or above the upper quartile are indicated by stars.

Means of individually determined IC50 values for the drug sensitivity measurements were compared between cases and controls using the Mann Whitney U test and also illustrated by Box-and-Whisker plots.

To assess the ability to discriminate between each case group and corresponding controls, a binary logistic model adjusted for age was fitted for each variable. For every model, the odds ratio (OR) for a one unit change with its 95% confidence interval (CI) and the corresponding *P* value are given. In addition, we performed receiver operation characteristic (ROC) curve analysis, and the area under the ROC curve (AUC) is presented together with the corresponding *P* value.

Associations of DSB repair frequencies, PARP activities or drug sensitivities with age were evaluated using the non-parametric Spearman rank correlation coefficient r_s_ and illustrated using scatter plots.

Statistics are summarized as percentages for categorical variables and as means ± standard deviation (SD) for continuous variables.

All statistical analyses described above were performed with IBM SPSS Statistics, Version 22.0 software (IBM Corp, Armonk, NY). GraphPadPrism 5.04 software (La Jolla, USA) was used to calculate IC50 values and to test for statistically significant differences of IQD- and carboplatin-response curves between cases and controls using Extra sum-of-squares F-test. All *P* values are two-sided and *P* values < 0.05 are considered statistically significant; there was no adjustment of the significance level for multiple comparisons.

## SUPPLEMENTARY MATERIALS FIGURES AND TABLES


