# Supplementary material for: Increased single-strand annealing rather than non-homologous end-joining predicts hereditary ovarian carcinoma
Source: Oncotarget. 2017 Oct 9;8(58):98660–76. doi: 10.18632/oncotarget.21720 (PMC5716758; doi:10.18632/oncotarget.21720)
Supplement: Supplementary file 1 [file oncotarget-08-98660-s001.pdf]

Increased single-strand annealing rather than non-homologous end-joining predicts hereditary ovarian carcinoma

SUPPLEMENTARY MATERIALS

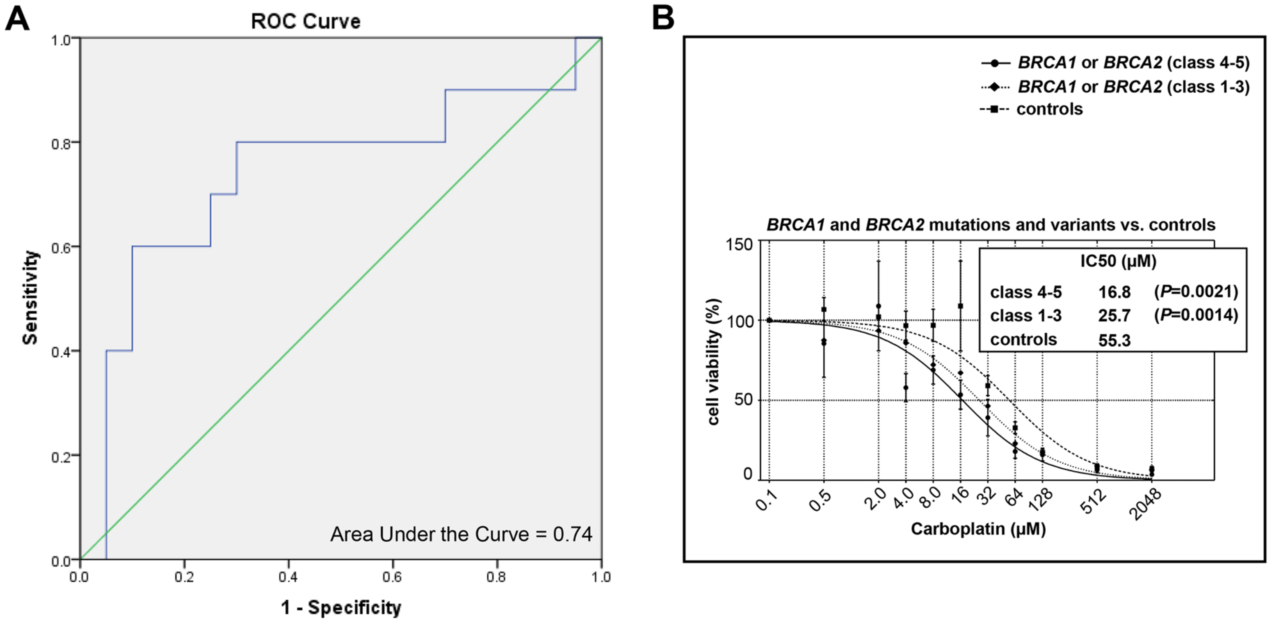

Supplementary Figure 1: Comparison of high-risk individuals with pathogenic *BRCA1* and *BRCA2* mutations and controls. (A) ROC curve of SSA values for PBLs derived from high-risk individuals with a pathogenic (class 5) or likely pathogenic (class 4) *BRCA1* and *BRCA2* mutation (n=10) and controls (n=20), adjusted for internal standard and log_{10} transformed. Area under the curve: 0.74, *P*=0.035. **(B)** Cell viabilities following carboplatin treatment were assessed by use of MTT assay after 48h of carboplatin treatment (as described in the legend to Figure 5). Mean survival curves are graphically presented for PBLs derived from high-risk individuals with a pathogenic (class 5) or likely pathogenic (class 4) *BRCA1* and *BRCA2* mutation (n=6), with *BRCA1* or *BRCA2* variants of uncertain significance (class 3), likely benign (class 2) and benign (class 1) variants (n=15) versus controls from the corresponding age subgroup of >=30 and <65 years (n=16). Calculation of IC50 values and statistical tests for differences between survival curves were performed using GraphPad Prism software. The survival curve for matched controls is the same one as displayed in Figure 5B versus all high-risk individuals. The mean survival curves for carriers of class 4-5 *BRCA1* or *BRCA2* mutations as well as class 1-3 variants are significantly different from the survival curve of controls (*P*=0.0021 and *P*=0.0014, respectively), whereby the survival curves for class 4-5 *BRCA1* or *BRCA2* mutations and class 1-3 variants show a trend towards a statistical discrimination (*P*=0.0842).

Supplementary Table 1: Characteristics of all study participants [a]

	High-risk individuals		Controls [b]		Patients	
	**n**	**Mean (SD)** [c]**/ percentage**	**n**	**Mean (SD)/ percentage**	**N**	**Mean (SD)/ percentage**
**Age**	38	45.6 (7.7)	34	61.0 (11.9)	40	61.0 (13.7)
**Age distribution**						
30-39	9	24%	1	3%	3	8%
40-49	16	42%	7	20%	6	15%
50-59	12	32%	6	17%	11	28%
60-69	1	3%	10	29%	6	15%
70-79	0	0%	8	23%	11	28%
>=80	0	0%	2	6%	3	8%

[a] PARP activities were tested in all high-risk individuals listed here in Supplementary Table 1 and all but one ovarian cancer patient and all but one control. DSB repair activities and drug sensitivities were tested in subgroups thereof (see Table 1).
[b] Note that for the comparison between high-risk individuals and controls, a control subgroup was analyzed encompassing the corresponding age group of >=30 and <65 years. For the comparison between patients and controls, a control subgroup was analyzed encompassing the corresponding age group of >35 years (see Table 1).
[c] SD = standard deviation.

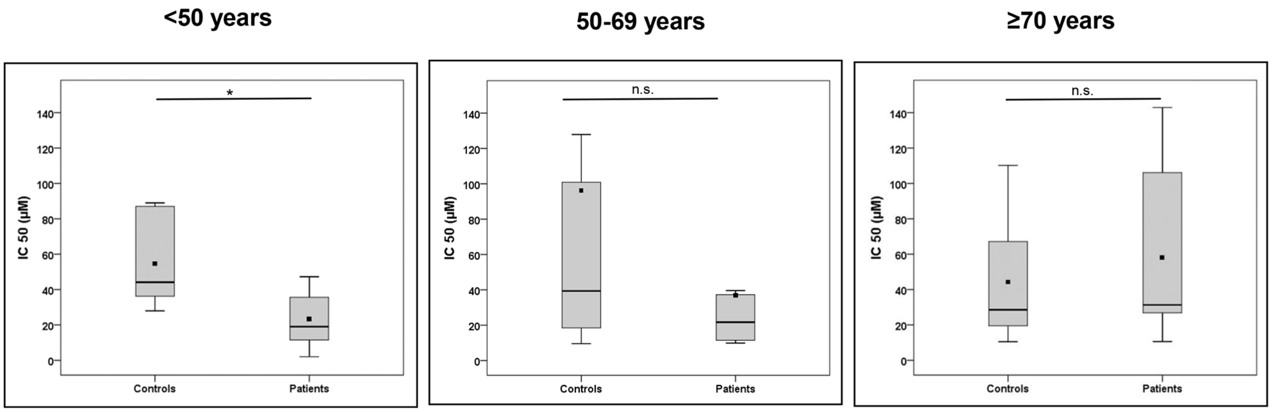

Supplementary Figure 2: Increased Carboplatin sensitivity in patients with early-onset ovarian cancer. Cell viabilities following Carboplatin treatment were assessed by use of MTT assay as described in the legend to Figure 5. Mean IC50 values were calculated for each ovarian cancer patient and control (>35 years) and compared using the Mann Whitney U test for different age subgroups, namely age <50 (controls: n=6, IC50=54.7µM; patients, n=7, IC50=23.2µM; P=0.046), age 50-69 (controls: n=13, IC50=96.0µM; patients, n=10, IC50=36.9µM; P=0.154) and age ≥70 (controls: n=8, IC50=43.9µM; patients, n=9, IC50=58.0µM; P=0.441). Please note that for the age group 50-69 years three outliers are not shown in the boxplot for scaling reasons (but were included in the statistical analysis). $^{*}P<0.05$; n.s., non-significant.

Supplementary Table 2: Differences between chemosensitivities of high-risk individuals versus controls and ovarian cancer patients versus controls

	High-risk individuals		Controls [a]		
**IC50 value**	**n**	**Mean (SD) µM**	**n**	**Mean (SD)µM**	***P* value [b]**
**IQD**	25	111.71 (118.0)	15	130.8 (91.4)	0.364
**Carboplatin**	29	29.0 (22.2)	16	66.5 (71.2)	0.035
	**Patients**		**Controls**		
**IC50 value**	**n**	**Mean (SD)µM**	**n**	**Mean (SD)µM**	***P* value**
**IQD**	23	90.4 (50.7)	25	114.6 (78.6)	0.516
**Carboplatin**	26	40.5 (43.2)	27	71.4 (93.2)	0.126

[a] Chemosensitivities were individually adjusted for mock-treated control (100%) analyzed in split samples each. Note that for the comparison between the case groups (high-risk individuals, patients) and controls, control subgroups were analyzed encompassing the corresponding age group each (controls compared with high-risk individuals: age >=30 and <65; controls compared with patients: age >35; for details of subgroups see Table 1).
[b] *P* values refer to comparisons of IC50 mean values per individual using the Mann Whitney U test.

Supplementary Table 3: Predictive power of IC50 values for carboplatin to discriminate between high-risk individuals and controls[a]

**IC50 value**	**High-riskindividualsn**	**Controls[b]n**	**OR[c] (95% CI)**	***P* value**	**Area under the ROC curve**	***P* value**
**Carboplatin**	29	16	0.963 (0.933-0.995)	0.021	0.69	0.035

[a] Chemosensitivities were individually adjusted for the mock-treated control (100%) analyzed in split samples each.
[b] As for the comparison between high-risk individuals and controls, the control subgroup was analyzed encompassing the corresponding age group >=30 and <65.
[c] The odds ratio (OR) per unit change given is based on age-adjusted binary logistic regression analysis. Please note that a unit change represents a change of the IC50 value by only 1 µM. An OR below 1 corresponds to a decrease of IC50 values in high-risk individuals.
